# Effectiveness of High-Power Laser Therapy via Shear Wave Speed Analysis on Pain and Functioning in Patients with Lateral Epicondylitis: A Proof-of-Concept Study

**DOI:** 10.3390/jcm13072014

**Published:** 2024-03-29

**Authors:** Nicola Marotta, Alessandro de Sire, Lorenzo Lippi, Lucrezia Moggio, Paolo Mondardini, Maria Sgro, Isabella Bartalotta, Roberta Zito, Teobaldo Giroldini, Marco Invernizzi, Umile Giuseppe Longo, Antonio Ammendolia

**Affiliations:** 1 Department of Experimental and Clinical Medicine, University of Catanzaro “Magna Graecia”, 88100 Catanzaro, Italy; nicola.marotta@unicz.it; 2 Research Center on Musculoskeletal Health, MusculoSkeletalHealth@UMG, University of Catanzaro “Magna Graecia”, 88100 Catanzaro, Italy; ammendolia@unicz.it; 3 Department of Medical and Surgical Sciences, University of Catanzaro “Magna Graecia”, 88100 Catanzaro, Italy; maria.sgro@studenti.unicz.it (M.S.); isabella.bartalotta@studenti.unicz.it (I.B.); roberta.zito@studenti.unicz.it (R.Z.); teobaldo.giroldini@studenti.unicz.it (T.G.); 4 Department of Scientific Research, Campus LUdeS Lugano (CH), Off-Campus Semmelweis University of Budapest, 1085 Budapest, Hungary; lorenzolippi.mt@gmail.com; 5 Rehabilitation Unit, Ospedale degli Infermi, 13875 Biella, Italy; lucrezia.moggio@aslbi.piemonte.it; 6 Department of Sport Science, Università di Bologna, 40100 Bologna, Italy; 7 Department of Health Sciences, University of Eastern Piedmont “A. Avogadro”, 28100 Novara, Italy; marco.invernizzi@med.unipo.it; 8 Translational Medicine, Dipartimento Attività Integrate Ricerca e Innovazione (DAIRI), Azienda Ospedaliera SS. Antonio e Biagio e Cesare Arrigo, 15121 Alessandria, Italy; 9 Research Unit of Orthopaedic and Trauma Surgery, Fondazione Policlinico Universitario Campus Bio-Medico, Via Alvaro del Portillo, 200, 00128 Rome, Italy; g.longo@policlinicocampus.it; 10 Research Unit of Orthopaedic and Trauma Surgery, Department of Medicine and Surgery, Università Campus Bio-Medico di Roma, Via Alvaro del Portillo, 21, 00128 Rome, Italy

**Keywords:** lateral epicondylitis, high-power laser therapy, shear wave velocity, ultrasonography, HILT

## Abstract

**Background**: Lateral epicondylitis (LE) causes lateral elbow pain due to the overuse of the common extensor tendon. Several therapies have been proposed for pain relief and functional recovery, including physical therapy, minimally invasive injection approaches, and physical agent modalities such as laser therapy. **Methods**: Our study evaluates the impact of high-power laser therapy (HPLT) on pain and functioning. The HPLT protocol consists of 10 daily sessions using a LASERIX PRO device. The healthy elbow of each participant was also considered as a control group. The outcomes assessed were the Numerical Rating Scale (NRS) for pain, QuickDASH questionnaire for functionality, and shear wave velocity (SWS) through ultrasonography. Assessments were conducted at baseline (T0), post-treatment (T1), and 2-week follow-up (T2). **Results**: Sixteen participants (81.2% male, mean age 40.4 ± 5.53 years) completed the study. Post-treatment, pain significantly decreased (NRS: T0 6.13 ± 0.96; T1 2.75 ± 1.69; *p* <  0.001), functionality improved (QuickDASH: T0 69.88 ± 10.75; T1 41.20 ± 3.78; *p* < 0.001), and shear wave velocity increased (SWS (m/s): T0 1.69 ± 0.35; T1 2.56 ± 0.36; *p* <  0.001). **Conclusions**: At the 2-week follow-up, pain relief was maintained, and shear wave velocity showed no further significant change. Shear wave velocity assessments might be considered a useful diagnostic tool. However, further research is needed to support the role of HPLT and shear wave velocity in the rehabilitation management of LE.

## 1. Introduction

Lateral epicondylitis (LE), also known as tennis elbow, is tendinosis of the extensor group muscles in the lateral elbow, related to the overuse of the common extensor tendon (CET), which arises from the muscle fibers used predominantly for wrist extension, as well as the extensor carpi radialis brevis tendon origin, which is most often the only area affected in LE [1,2,3]. LE is more common in individuals aged 40–45 years or people with manual labor occupations, with men and women being affected equally. Despite tennis players making up less than 10% of the patient population, half of the tennis players do develop pain around the elbow, more than three-quarters of which is characteristic of true “tennis elbow” [4]. The diagnosis of LE is mainly based on physical examination and pain scoring; nevertheless, imaging studies, including ultrasonography and Nuclear Magnetic Resonance (NMR) imaging, might support the diagnosis [5]. Although NMR imaging has higher sensitivity and specificity in the diagnosis of LE, ultrasonography is commonly used in clinical practice due to its low cost, great accessibility, and real-time evaluation [6]. In this scenario, Hong et al. [7] demonstrated that shear wave elastography (SWE) could be a valuable additional diagnostic tool for the diagnosis of LE, as it is able to evaluate the echostructure but also weigh the degree of stiffness of the tendinopathy. Shear wave propagation analysis allows the shear wave velocity (SWS) to be calculated through the impedance to propagation at different depths within the tissue [8]. In this context, in the management of LE, physical therapy is applied to increase functioning and reduce pain in order to accelerate the progression of recovery and help patients normalize their daily living activities [9]. The treatment modalities commonly used for tendinopathies are: pharmacological therapies, hyaluronic acid, injections, oxygen–ozone therapy, therapeutic exercise, manual therapy, orthoses, physical agent modalities (PAMs), such as ultrasound therapy, laser, extracorporeal shock wave therapy, and in the most severe cases, surgical approaches [10,11,12,13,14,15,16,17]. However, no consensus about the best treatment for improving pain and function in people with LE has been reached; nonetheless, among rehabilitative approaches, therapeutic exercise and physical therapy have reported positive effects on people with LE, regarding pain decrease and tendon strength enhancement [18]. Recently, El Meligie et al. [19] concluded that High Power Laser Therapy (HPLT) could improve grip strength, pain, and functional ability at midterm follow-up (5 to 26 weeks). As a matter of fact, with a high-power laser approach, it is possible to reach and stimulate larger and/or deeper areas; consequently, more energy could be transferred into tissues compared to low-level laser therapy (LLLT) [20,21]. On the other hand, to date, clinical improvement data have not been underlined instrumentally.

Therefore, the aim of this study was to weigh the effectiveness of HPLT on pain relief, functioning, and improvement of muscle stiffness through shear wave velocity in people affected with LE.

## 2. Materials and Methods

### 2.1. Participants

This cohort clinical study was assessed and recorded by the Local Ethics Committee (“Comitato Etico Territoriale Regione Calabria”), providing the following code: 259/2019, in accordance with the Declaration of Helsinki and following the ethical guidelines of the responsible legislative institute. The study protocol followed the STROBE guidelines and was performed at the Physical and Rehabilitative Unit of the University Hospital “Renato Dulbecco” of Catanzaro, Italy, where patients were recruited from October 2022 to October 2023. Written informed consent was obtained from all participants for treatment and data processing.

Subjects for the study group with a diagnosis of lateral epicondylitis (the LE group) were included with an age ranging between 18 and 65 years, with unilateral elbow pain for at least 2 weeks, and with the following clinical characteristics: (1) no other upper limb disorders (e.g., cervical radiculopathy, carpal tunnel syndrome); (2) minimal pain at rest with a Numerical Rating Scale (NRS) ≥ 4; (3) positive palpation test of the lateral epicondyle; (4) ultrasound (US) imaging showing a positive report for thickening and an echo-structure suggestive of common extensor tendinopathy. Nonetheless, we set the following exclusion criteria: (1) patients receiving regular painkillers; (2) intra-articular or peri-tendinous injections of steroids or hyaluronic acid in the previous six months; (3) physical therapy in the previous two months; (4) confirmed rupture of the tendon; (5) previous elbow surgery; (6) radial tunnel syndrome. Additional exclusion criteria were the presence of contraindications to PAM application; lastly, patients who had skin disorders or lesions near the area of PAM administration. Furthermore, for each participant included in this study, we also considered the healthy elbow as a control group (CNT group).

### 2.2. Intervention

**Figure 1:**
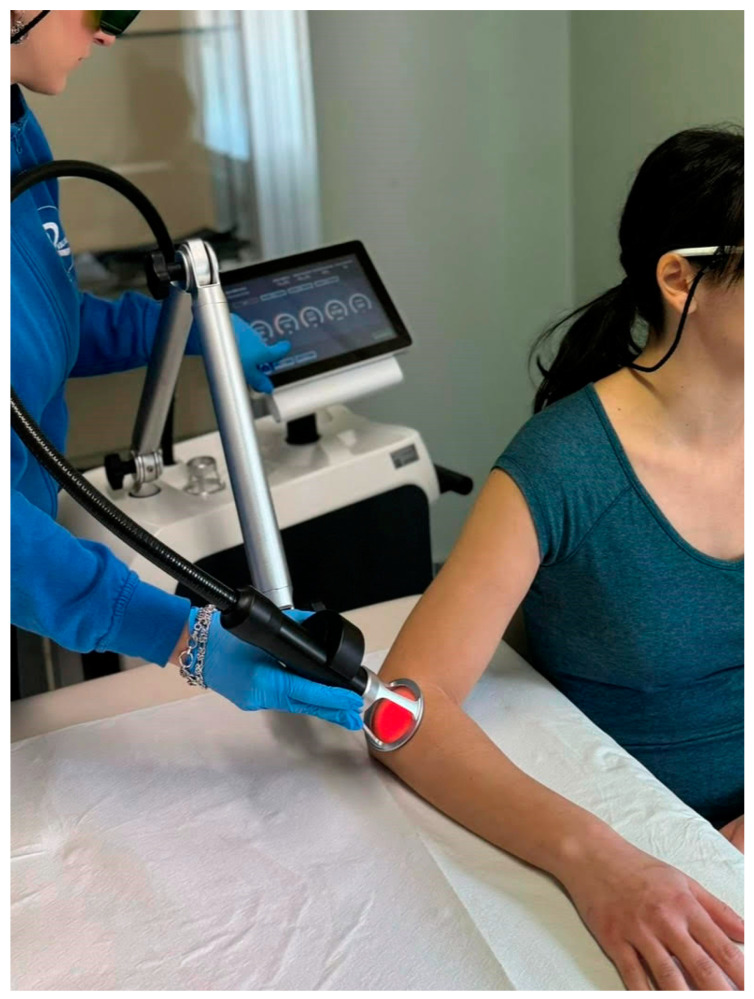
Application of laser therapy via emitter spacer.

The HPLT program involved 10 daily sessions (five per week, for 2 weeks), via a LASERIX PRO device (GN med, 40026 Imola (BO), Italy), applied using a fixed probe with a 30 mm spacer, as depicted in Figure 1.

Each HPLT session had a 13 min time of administration, divided into three phases. Firstly, a 7 min session of 226 Joules of energy (18 Khz, peak power of 600 W) was delivered; secondly, a 3 min administration of 226 Joules of energy, albeit with a lower frequency of 14 Khz and a peak power of 900 W; thirdly, a final phase lasting 3 min, again with 226 Joules of energy, with a frequency of 10 Khz and a peak power of 1200 W was applied [22].

### 2.3. Outcome Measures

Participants’ perceived pain was rated at each timepoint using a numerical rating scale (NRS), requiring patients to rate their pain on a defined 0–10 scale, where 0 is no pain, and 10 is the worst pain imaginable. Furthermore, the Quick Questionnaire on Disabilities of the Arm, Shoulder, and Hand (QuickDASH) was used to assess the subjects’ disability and functioning. The QuickDASH is a self-administered questionnaire that measures the physical functions and symptoms of patients with upper limb problems through 11 items, where each of them includes five response options, providing a total score on the scale from 0 to 100, where 0 indicates no disability, and higher scores up to 100 or more indicate severe disability. The first eight items of the QuickDASH questionnaire measure the patient’s daily living functions and limitations in social activity. The last three elements consider, respectively, the intensity of pain, sensation of “pins and needles”, and finally, the possible impairment of sleep due to pain. Ultrasonographic examination of the patients was performed before treatment (T0), at the end of treatment (T1), at a follow-up 2 weeks after the end of treatment (T2) by a clinician with 4 years of experience in musculoskeletal ultrasonography, who was blinded to the patients’ clinical data. The patient sat in front of the examiner with the elbow flexed at 90° and the thumb up. Ultrasonographic examination was performed using an Aplio A (Canon Medical Systems Europe, Zoetermeer, The Netherlands) ultrasound machine with an 18–6 MHz linear array transducer. The ultrasonographic technique used in a previous study evaluating lateral epicondylitis with ultrasonography was accepted as a reference [7,8]. Ultrasonographic examination of the common extensor tendon, obtained on a longitudinal plane, provides elastograms, which can be graduated by color or elasticity of the tissue within the regions of interest, ranging from blue to red, representing the softness and hardness of the tissue, respectively, via Shear Wave Elastography (SWE) [8]. Then, the integrated SWE elastograms automatically displayed SWS, and Young’s modulus and shear wave speed (SWS) were the primary measurements used for the analysis [7]. The cut-off value of healthy SWS was set ≥2.31 (90.91% sensitivity and 100.0% specificity) [7].

### 2.4. Statistical Analysis

Statistical analysis was performed via jamovi (version 2.2.6). We measured data for normal distribution via the Shapiro–Wilk test, and Leven’s test was used for assessing variance analysis homogeneity. Categorical or dichotomous data were defined with frequencies, while continuous data were described as means ± SD. Effect sizes were definied via Cohen’s d expression with a 95% CI. Effect sizes were categorized as trivial < 0.5; suitable between 0.5 and 0.8; and large > 0.8. For each test, statistical analyses were two-tailed, and a *p*-value cut-off set at <0.05 was considered significant [23]. All outcomes were analyzed for within-group and between-group comparisons across different time points. The G-Power statistics module from jamovi software (2.4.14) was used to estimate the proper sample size, considering an alpha level of 0.05 with 80% power and a minimum effect size of 0.40. Through a repeated-measures analysis of variance of group relations, a fit sample size was established to be 15.

## 3. Results

**Figure 2:**
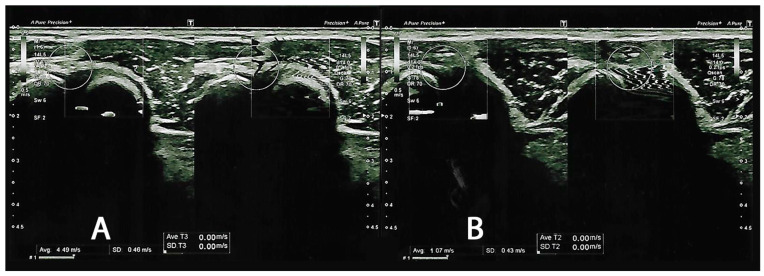
Shear wave velocity elastograms. (**A**) depicts a frame of the healthy lateral common extensor tendon (CNT), reporting a shear wave velocity of 4.49 m/s. (**B**) represents a picture of lateral epicondylitis (LE), reporting a shear wave velocity of 1.07 m/s.

In total, sixteen subjects (13 males and three females) who met the study eligibility criteria were included and observed in the follow-up assessment. The mean age of the participants was 40.4 ± 5.53 years (min: 29, max: 51). Moreover, we reported a BMI of 26.8 ± 2.51 (min: 23.9 kg/m^2^, max: 33.1 kg/m^2^). We reported no dropouts from the study, nor did we report any adverse effects, even at the 4-week follow-up. As reported in Figure 2, we reported how we evaluated the elastograms for the healthy sides and the sides affected by LE, demonstrating the respective SWSs.

During the baseline subject assessment, we reported that the LE side group had a noticeable severity of pain (NRS: 6.13 ± 0.96), compared to the healthy CNT side (NRS: 0.69 ± 0.79). In parallel, we reported a difference in SWS (CNT: 3.84 ± 0.51 versus LE: 1.69 ± 0.35), with results comparable to the study of Hong et al. [7]. At T1, all outcomes for the side affected by LE improved significantly, as reported in Table 1.

**Figure 3:**
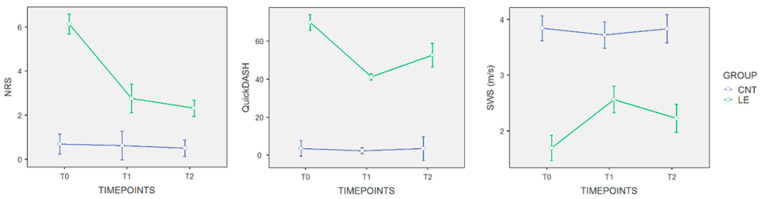
Repeated measures plot for study outcome. Blue lines: CNT, healthy elbow group; green lines: LE, lateral epicondylitis group. Starting from T1 and continuing through the 2-week follow-up at T2, the treatment program maintains the pain relief results obtained (NRS for LE, T1: 2.75 ± 1.69 versus 2.31 ± 0.87 at T2, ΔT1-T2: *p* = 0.311) but does not yield further reductions in terms of disability (QuickDASH for LE, T1: 41.20 ± 3.78 versus 52.71 ± 16.96 at T2, ΔT1-T2: *p* = 0.021). Conversely, stiffness of the common extensor tendon shows a statistically non-significant difference (*p* = 0.065) at the end of the treatment (SWS for LE, T1: 2.56 ± 0.36) compared to the 2-week follow-up (SWS for LE, T2: 2.23 ± 0.24).

However, starting from T1 and continuing through the 2-week follow-up at T2, the treatment program maintained the results obtained for pain relief (NRS for LE, T1: 2.75 ± 1.69 versus 2.31 ± 0.87, ΔT1-T2: *p* = 0.311), but did not result in further decreases in terms of disability (QuickDASH for LE, T1: 41.20 ± 3.78 versus 52.71 ± 16.96, ΔT1-T2: *p* = 0.021). On the other hand, stiffness of the common extensor tendon reported an SWS not significantly different (*p* = 0.065) at the end of the treatment (SWS for LE, T1: 2.56 ± 0.36) compared to the 2-week follow-up (SWS for LE, T2: 2.23 ± 0.24), as depicted in Figure 3.

## 4. Discussion

The aim of the proof-of-concept study was to evaluate the effectiveness of HPLT on pain relief, improvement of muscle stiffness through shear wave velocity assessment, and functioning in people with LE. Main findings showed that the HLPT program sustained the pain decrease outcomes found (NRS for LE, T1: 2.75 ± 1.69 versus 2.31 ± 0.87 at T2, ΔT1-T2: *p* = 0.311) but does not produce further reductions in terms of disability (QuickDASH for LE, T1: 41.20 ± 3.78 versus 52.71 ± 16.96 at T2, ΔT1-T2: *p* = 0.021), starting at the end of treatment and ongoing over the 2-week follow-up at T2. Contrariwise, common extensor tendon stiffness reported a statistically non-significant variance (*p* = 0.065) at T1 with an SWS of 2.56 ± 0.36 versus the T2-SWS of 2.23 ± 0.24.

To the best of our knowledge, no clinical studies have explored the impact of HPLT administration on common extensor tendon stiffness in patients with LE. LE, known as “tennis elbow”, is the most common cause of lateral elbow pain due to the overuse of the common extensor tendon; overload and detraining might be catabolic insults for tendon tissue, consequential to the synthesis of collagenase, proteinase, and pro-inflammatory cytokines [22]. Nowadays, complementary imaging is required to assist in diagnosis; hence, elastography is gradually attracting public attention [8]. Augmented tendon compressibility, denotative of tendon softening, is deemed a further sonographic sign of common extensor tendinopathy [24].

Consequently, elastographic tendon softening could be a valuable approach for the differential diagnosis of people with LE, just as was the case in the group affected by LE; indeed, HLPT was able to provide pain relief, and also improve the echostructure of the tendon at the end of treatment and then maintain it at follow-up. Lee et al. [25] estimated the thickness of the common extensor in patients with LE, reporting it to be quite thicker than in healthy control subjects. This phenomenon may be explained by the swelling rendered by morbid alteration CET in LE, leading to an increase in thickness. Nonetheless, considerably overgrown collagen fibers contributing to the raising of the thickness of normal CET presumably result in stiffer elasticity [26].

To date, different authors have sustained the use of eccentric exercise to improve pain and functioning in people with LE. In comparison with concentric conditioning, eccentric exercise has exhibited a noteworthy decrease in self-perceived pain [27]. Eccentric contraction might seem to facilitate tendon cell proliferation, generating augmented collagen cross-linking and decreased neuro-vascular ingrowth, which may modulate pain pathways [28]. Nevertheless, the association between the conditioning model and pain remains undefined in lateral epicondylitis, and it is constantly argued whether eccentric exercises should be executed with pain. Additional rehabilitative procedures have been currently presented for LE pain recovery, including injections of different agents such as adipose-derived mesenchymal stromal cells, botulinum neurotoxin type A (BoNTA), platelet-rich plasma (PRP), and hyaluronic acid (HA) [29,30,31].

Furthermore, in the scenario of rehabilitation approaches for LE management, physical agent modalities play a key role in clinical practice. In particular, laser therapy appears to be useful in distinct musculoskeletal disorders by decreasing edema and inflammation, managing pain, and stimulating tissue recovery [32]. Nevertheless, controversial evidence is obtainable concerning the advantages of LLLT in patients with common extensor tendinopathy, and only short-term pain relief was documented [33,34]. Although evidence on HPLT is scarce, some observational studies indicate the usefulness of this PAM in LE for pain and disability management, considering its analgesic and regenerative impacts, attributable to its ability to determine a slow and small absorption of light from part of the chromophores, thus diffusing light in all directions, causing the “scattering” phenomenon. This phenomenon induces tissue stimulation (photobiological effect) and increases mitochondrial oxidative reactions and the production of DNA, RNA, or adenosine triphosphate (photochemical effect) [35].

In this study, the common extensor SWS reported consistent results in aiding the diagnosis of LE, especially when compared to the healthy side of the participants. In fact, the SWS of elbows affected by LE was lower than the SWE in healthy elbows and increased after treatment [8]. The elastrographic evaluation of the SWS of the CET appears reliable in this study and remains a reproducible technique accessible in clinical practice [24]. Finally, SWS could become a valid imaging method for LE and could help monitor the therapeutic effect of physical agent modalities [7]. Furthermore, it could provide instrumental proof that the approach with HPLT is also capable of improving the chronic tendinopathic picture of subjects affected by LE from an echo-structural point of view [36,37].

However, our study has some limitations. First, the retrospective design as well as the lack of two-arm allocation might limit certain conclusions. Second, our included participants mat not be representative of the general population affected by LE, including elderly people, obese people, and workers involved in repetitive and strenuous activities. Lastly, a larger sample size will be necessary to provide significantly generalizable results, especially to support reliable shear wave speed diagnostics. Future clinical trials are needed to confirm the positive outcomes of this proof-of-principle study endorsing the effectiveness of such high-power physical agent modalities in the management of LET.

## 5. Conclusions

In conclusion, our findings support the use of HPLT as an adjunct to a multimodal approach, providing effects in terms of pain relief, reducing echo-structure alterations and stiffness, and increasing SWS in patients with LE. In this scenario, US examination, particularly the use of SWS, might be a useful diagnostic tool for the management of common extensor tendinopathy. Despite this, these encouraging results require further studies with larger samples and randomized controlled study designs to demonstrate the effectiveness of PAMs, particularly HPLT, using shear wave speed analysis on functioning in patients with LE.

## Figures and Tables

**Table 1:** Within-group differences in the outcome measures for lateral epicondylitis and control groups.

		*T0*		*T1*		Δ*T0-T1*	*T2*		Δ*T1-T2*
	Group	Mean	SD	Mean	SD	p Value	Mean	SD	p Value
*QuickDASH*	CNT	3.53	3.09	2.25	2.32	*0.067*	3.38	2.61	*0.089*
	LE	69.88	10.75	41.20	3.78	***<0.001***	52.71	16.96	***0.021***
*NRS*	CNT	0.69	0.79	0.63	0.62	*0.766*	0.50	0.52	*0.531*
	LE	6.13	0.96	2.75	1.69	***<0.001***	2.31	0.87	*0.311*
*SWS (m/s)*	CNT	3.84	0.51	3.72	0.55	*0.877*	3.83	0.65	*0.589*
	LE	1.69	0.35	2.56	0.36	***<0.001***	2.23	0.24	*0.065*

CNT: healthy elbow group, LE: lateral epicondylitis group, NRS: Numerical Rating Scale, SWS: Shear Wave Speed.

## Data Availability

The dataset is available on request.
